# Recent advances in nonprotein amino acids: insights from function to biosynthesis

**DOI:** 10.1186/s40104-026-01396-w

**Published:** 2026-05-01

**Authors:** Yuhong Zou, Xi Jiang, Na Li, Shasha Zhong, Shimin Zhang, Yuanqing Ji, Haitao Yu, Xiangfang Zeng, Aihua Deng, Shiyan Qiao

**Affiliations:** 1 State Key Laboratory of Animal Nutrition and Feeding, Ministry of Agriculture and Rural Affairs Feed Industry Centre, College of Animal Science and Technology, China Agricultural University, Beijing, 100193 China; 2 Beijing Bio-Feed Additives Key Laboratory, Beijing, 100193 China; 3 Frontier Technology Research Institute of China Agricultural University in Shenzhen, Shenzhen, 518116 China

**Keywords:** Biomanufacturing, Classification, Metabolic engineering, Next-generation bioproducts, Nonprotein amino acids, Synthetic biology

## Abstract

The systematic exploration of novel bioactive compounds with superior functional properties is critical for driving innovations in agriculture, healthcare, and related fields, thereby becoming essential for advancing sustainable biotechnological solutions. Nonprotein amino acids (NPAAs), functional amino acids not incorporated into proteins, exhibit unique physiological activities and provide distinctive advantages in nutritional enhancement, functional product formulation, and food/feed processing. These attributes challenge the conventional perception of proteins as mere nutritional carriers, positioning NPAAs as promising bioproducts for biosynthesis and functional applications in agriculture, food, and medicine. This review summarizes the classification of the available NPAAs based on their synthetic substrates for the first time and then outlines their diverse functional roles. A comprehensive analysis of recent advances in biosynthetic pathways, engineering strategies, and production level demonstrates their primary research progress in the laboratory phase. The further sustainable biomanufacturing of NPAAs is hampered by several challenges, including poorly elucidated biosynthetic mechanisms, limited robustness and low productivity of microbial strains, and difficulties in scaling up production for industrial applications. Addressing these bottlenecks will require innovative strategies and technologies to facilitate the translation of NPAA production from bench to industry. This review offers valuable insights into the potential of NPAAs in the development of next-generation bioproducts of nutrition, immune regulation, antioxidant defense, and intestinal homeostasis maintenance, suggesting a promising direction for microbial production of high-performance bioactive molecules in agricultural synthetic biomanufacturing.

## Introduction

Systematic research and utilization of high-performance bioactive molecules are pivotal for meeting the growing demands of human livelihood and ensuring the long-term, stable development of agriculture, medicine, and interconnected industries. In traditional nutrition science, proteins are essential molecules in all living organisms, composed of 20 natural amino acids. These protein amino acids are crucial for various metabolic processes, including nutrition, immune regulation, antioxidant defense, and intestinal homeostasis maintenance. However, the limited diversity of these natural amino acids makes it challenging to meet the diverse structural and functional needs of proteins in life science [1].

Recent research has uncovered a special class of “nonprotein amino acids (NPAAs)” that are reshaping our understanding of amino acids. Different from traditional amino acids, they are not encoded by the genetic code, such as γ-aminobutyric acid, L-ornithine, and taurine, exist as free or small peptides within cells or tissues [2]. Over 700 kinds of NPAAs have been discovered, they play crucial roles in the growth, metabolism, structure, and function of living organisms. Due to their diverse structures, they greatly extend the function of amino acids. They demonstrate distinctive value in food processing, nutritional enhancement, and the development of functional products, surpassing the capabilities of traditional proteins. Especially, many NPAAs (like L-dopa, L-canavanine, etc.) have great potential in the treatment of various diseases and the field of alternative antibiotics. NPAAs do not contribute to protein composition but possess unique biological functions and significant application value in agriculture, medicine, food, and other fields [3].

**Fig. 1:**
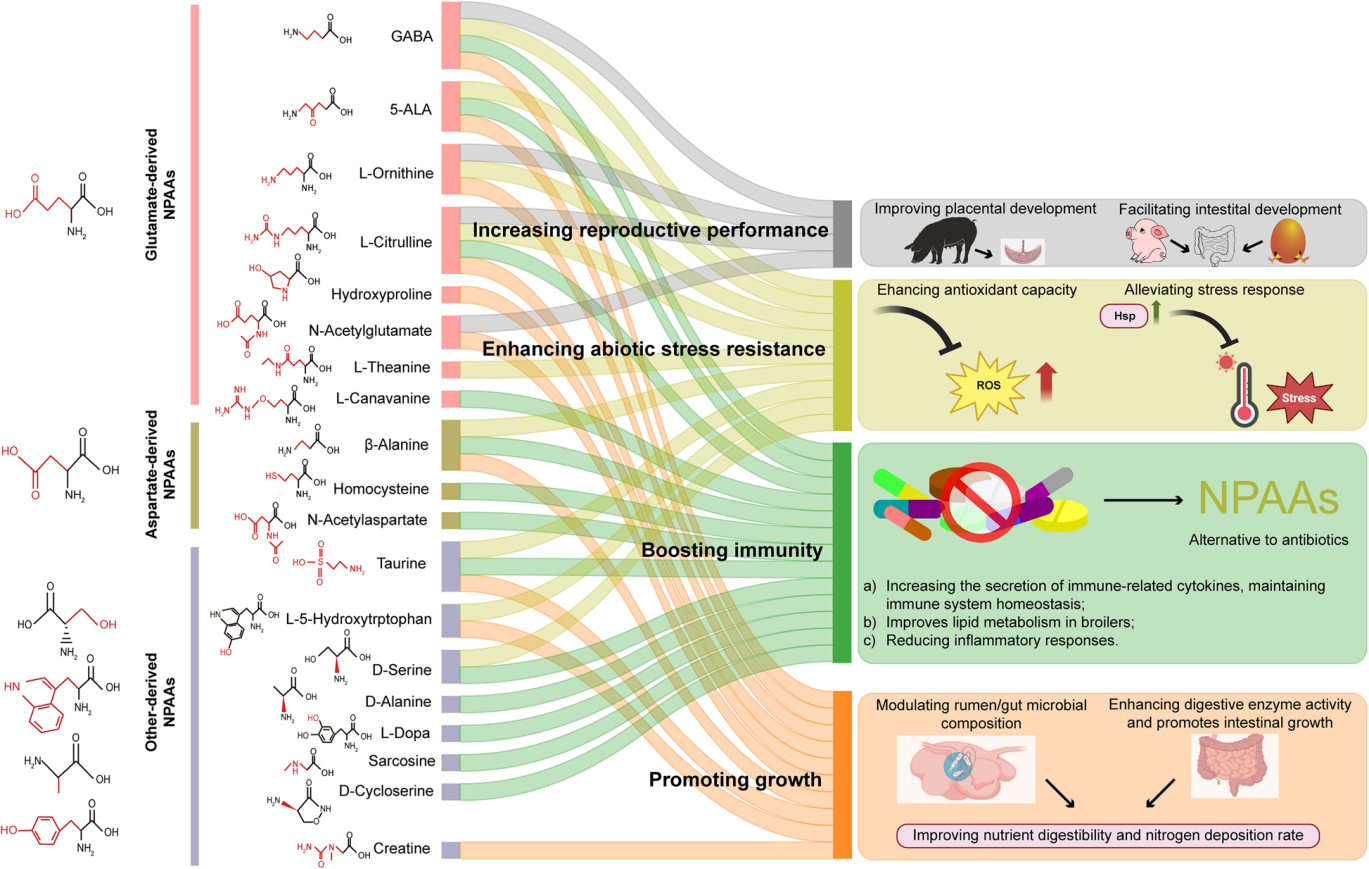
Overview of various functions of nonprotein amino acids. The glutamate-derived NPAAs, aspartate-derived NPAAs and other-derived NPAAs are shown as pink, khaki and purple boxes, respectively. Different functions are shown as different colored strips. Highlight the variations of the R groups from standard amino acids to NPAAs in red. Hsp, heat shock protein

The biosynthesis of NPAAs involves complex pathways, primarily including the following ways: (a) modification of standard amino acid synthetic pathways, such as hydroxylation and oxidative decarboxylation; (b) accumulation of intermediates from standard amino acid metabolism; and (c) racemization of amino acids [4]. These biosynthetic processes are closely related to protein amino acids. Based on their origins, NPAAs can be categorized into those derived from a specific protein amino acid. Figure 1 shows the classification of NPAAs based on the biosynthetic pathway for the first time, which have been classified into glutamate-derived NPAAs, aspartate-derived NPAAs, and other-derived NPAAs.

In 2023, the global amino acid market has reached 11.4 million tons and is expected to grow to 16.8 million tons by 2032 [5, 6]. Converting amino acids to NPAAs can address the overcapacity issue and extend the function of amino acid products. For example, the global GABA and β-alanine market was valued at tens of millions of dollars in 2025 [7, 8]. Furthermore, the global 5-ALA market was valued at 243 million dollars in 2025 and is forecast to reach a readjusted size of 409 million dollars by 2032 [9]. These NPAAs were widely used in agriculture, medicine, cosmetic and food fields. Currently, NPAAs can be produced through the method of chemical synthesis or biosynthesis [10, 11]. Chemical synthesis, while it is relatively well-established and capable of achieving high yields, often involves complex chemical reactions under harsh conditions (such as high temperature and high pressure) and generates toxic by-products, which could lead to environmental pollution [12, 13]. In contrast, biosynthesis, particularly microbial production, offers a straightforward, environmentally friendly with potential for reduced production costs [14]. This aligns well with the concept of “green and healthy” development and has received extensive attention.

In this review, we emphasized the important functions of NPAAs and summarized recent progresses in the biosynthesis of NPAAs, which became a superior method over chemical synthesis. Despite significant progress, their biosynthesis technology remains in its early stage, requiring continued innovation in metabolic engineering and synthetic biology approaches. We comprehensively summarize the current status of biosynthesis for NPAAs and their value in agricultural application, providing guidance for valuable insights for exploring a broader spectrum of high-performance bioactive compounds.

## The classification and function of NPAAs

Nonprotein amino acids, with their specific physiological activities, offer promising applications in agriculture, medicine, food, and cosmetics. The following sections summarize the functions and potential applications of NPAAs (Fig. 1).

### The classification of NPAAs

In this review, we classified NPAAs into glutamate-derived NPAAs, aspartate-derived NPAAs, and other-derived NPAAs, according to their biosynthetic pathway. As shown in Fig. 1, the conversion of standard amino acids to NPAAs involves one or more reaction steps, involves amidation reaction, acetylation reaction, dicarboxylic reaction, hydroxylation reaction, or racemization reaction, etc.

For example, the conversion of glutamate to GABA and L-theanine include only one step, -CH_2_-CH_2_-CH_2_-NH_2_ or -CH_2_-CH_2_-CONH-CH_2_-CH_3_ is synthesized from the R group of glutamate (-CH_2_-CH_2_-COOH) by losing -COOH or condensation with ethylamine. Different from the biosynthesis of GABA, the dicarboxylic reaction from aspartate to β-alanine, occurs on the α-carboxyl group rather than on the R group. Notably, from glutamate to N-acetylglutamate, the acetylation reaction occurs on the α-amino group rather than on the R group. The α-amino group of glutamate (-NH_2_) reacts with acetyl coenzyme A to form -NH-CO-CH_3_. Similarly, N-acetylaspartate is synthesized from aspartate by acetylating the α-amino group. Furthermore, the conversion of tryptophan to L-5-hydroxytryptophan and the conversion of tyrosine to L-dopa also involves only one step hydroxylation reaction. D-Alanine and D-serine is able to biosynthesize from L-alanine or L-serine through racemization reaction catalyzes by racemase.

However, the conversion of glutamate to 5-ALA, L-ornithine, etc. involves many reactions, the variation of R group is diversity. For example, the biosynthesis of L-ornithine from glutamate involves three major transformations of the R group: (1) -CH_2_-CH_2_-COOH is reduced to -CH_2_-CH_2_-CHO; (2) -CH_2_-CH_2_-CH_2_OH is synthesized from -CH_2_-CH_2_-CHO through transamination; (3) finally transfers to -CH_2_-CH_2_-CH_2_-NH_2_.

### The function of NPAAs

#### Increasing reproductive performance

In animal husbandry, it is well-known that dietary arginine supplementation is beneficial to reproductive performance. Consequently, as important precursors, L-ornithine, L-citrulline and N-acetylglutamate are reported to be potential in increasing reproductive performance. Dietary L-ornithine supplementation with appropriate concentration for gestating sows facilitates the intestinal development of piglets [15]. As a dietary supplement, L-citrulline and N-acetylglutamate were demonstrated to be able to improve the semen quality [16, 17]. In addition, Gemmel et al. [18] suggested the potential of L-citrulline as a therapeutic in treating preeclampsia, a spontaneously occurring pregnancy complication, by improving nitric oxide-mediated vascular function. Furthermore, L-citrulline and GABA have also been demonstrated to enhance reproductive outcomes in female animals by improving placental development and survival [19, 20].

#### Enhancing abiotic stress resistance

In livestock and poultry production, prolonged stress from environmental factors like temperature and feeding density can adversely affect production performance and increase disease incidence. However, research has shown that certain NPAAs can bolster stress resistance in animals.

GABA, as an important inhibitory neurotransmitter, plays a vital role in reducing stress responses. It increases neuronal membrane permeability while facilitating chloride ion flux, subsequently cellular hyperpolarization. This hyperpolarization mediates postsynaptic inhibition, which in turn attenuates physiological stress responses [21]. Chen et al. [22, 23] found that GABA effectively alleviates the heat stress in beef cattle by increasing antioxidant capacity and improving blood homeostasis by stimulating the expression of heat shock proteins. Feeding studies have demonstrated that the appropriate addition of GABA to the basic diet of livestock and poultry diets can counteract production declines caused by high temperature and dense feeding conditions, thereby alleviating stress response [22–27]. Furthermore, L-citrulline, L-theanine, and taurine were also reported to be promising additives in reducing negative effects caused by heat stress [28, 29]. In addition, GABA serves as a functional feed additive with the capability to alleviate placental oxidative stress in gestating sows [30]. Similarly, as a dietary supplement, L-theanine can enhance the intestinal mucosal barrier and antioxidant capacity to alleviate acute oxidative stress in white feather broilers [31].

#### Boosting immunity

With the advancements of large-scale breeding practices, disease has become a significant limiting factor in the breeding industry. Studies have shown that the addition of NPAAs like GABA and taurine to the diet is able to improve the immunity of livestock and poultry.

Nájera-Martínez et al. [32] evaluated the effects of different concentrations of GABA on the immune system of Nile tilapia using a basal level of 4.8 μg GABA per 100 g fish. The findings revealed that different concentrations of GABA significantly increase the secretion of immune-related cytokines in Nile tilapia, maintaining immune system homeostasis.

Taurine plays a significant role in the regulation of metabolic diseases. Zheng et al. [33] found that taurine played an essential role in maintaining the intestinal barrier integrity and inhibiting intestinal inflammation in mice with experimental colitis. Han et al. [34] showed that different dietary taurine supplementation improves lipid metabolism in broilers. Furthermore, taurine is also actively involved in reducing inflammatory responses and tissue damage [35, 36].

5-ALA, L-citrulline, and β-alanine can also boost immunity in livestock and poultry production. Chen et la. [37] found that adding 60 mg/kg 5-ALA to the diet significantly increase the haem oxygenase-1 expression and inhibit the TLR4/NF-κB signaling pathway, thereby exerting anti-inflammatory and antioxidant effects against lipopolysaccharide-induced challenges. Ma et al. [38] demonstrated that 1% to 1.5% L-citrulline supplementation could enhance immune functions of broilers by decreasing the level of interleukin-1β and tumor necrosis factor alpha. Wang et al. [39] discovered that β-alanine supplementation improves intestinal morphology and barrier function in piglets while reducing inflammatory responses.

#### Promoting growth

Dietary supplementation of NPAAs such as GABA (γ-aminobutyric acid), β-alanine, and taurine can promote the growth of animals. Ruenkoed et al. [40] demonstrated that addition of GABA to the Nile tilapia’s diet enhances digestive enzyme activity and promotes intestinal growth, improving nutrient absorption and overall growth. Similarly, several studies showed that the addition of hydroxyproline in aquatic feed was positive to fish growth performance [41, 42]. As the essential precursor of arginine, L-ornithine, L-citrulline and N-acetylglutamate are potential in improving growth performance. Wang et al. [43] revealed that amniotic injection of N-acetylglutamate improving early gut development and digestion capacity of broilers. In addition, L-citrulline was demonstrated to be useful in the modulation of growth performance, which is mostly associated with gut microbiota [38, 44]. Similar to L-citrulline, dietary 5-hydroxytryptophan supplementation improves growth performance of sheep by modulating rumen microbial composition [45]. Furthermore, 5-hydroxytryptophan as a dietary supplement was demonstrated to increase the average daily gain and decrease the diarrhea rate of weaned piglets [46]. Hu et al. [47] revealed that diets with appropriate β-alanine concentrations improve nutrient digestibility and nitrogen deposition rate, promoting the growth of beef steers. Shen et al. [48] found that taurine supplementation boosts the carbohydrate synthesis, protein digestion/absorption, and fat deposition of Nile tilapia, effectively promoting fish growth.

#### Other functions

Beyond the functions previously discussed, NPAAs exhibit a range of additional biological roles. For example, L-theanine and 5-hydroxytrptophan are able to improve sleep quality [49, 50]. L-Ornithine, as a dietary supplement, attenuates physical fatigue by promoting lipid metabolism and ammonia excretion [51]. Long-term L-citrulline supplementation was suggested to be a promising therapeutic intervention to anti-aging [52]. Taurine supplementation has been shown to prevent the decrease of superoxide dismutase to anti-aging [53]. A research by Nava-Gomez et al. [54] revealed that D-serine supplementation was able to reverse aging-associated brain alterations. Currently, 5-ALA, L-citrulline, β-alanine, taurine, and creatine are explored to improve exercise capacity [55–59]. These findings reveal the potential of NPAAs as functional components in healthcare, food, and feed products, capable of mediating specific physiological effects.

## Biosynthesis of glutamate-derived NPAAs

**Fig. 2:**
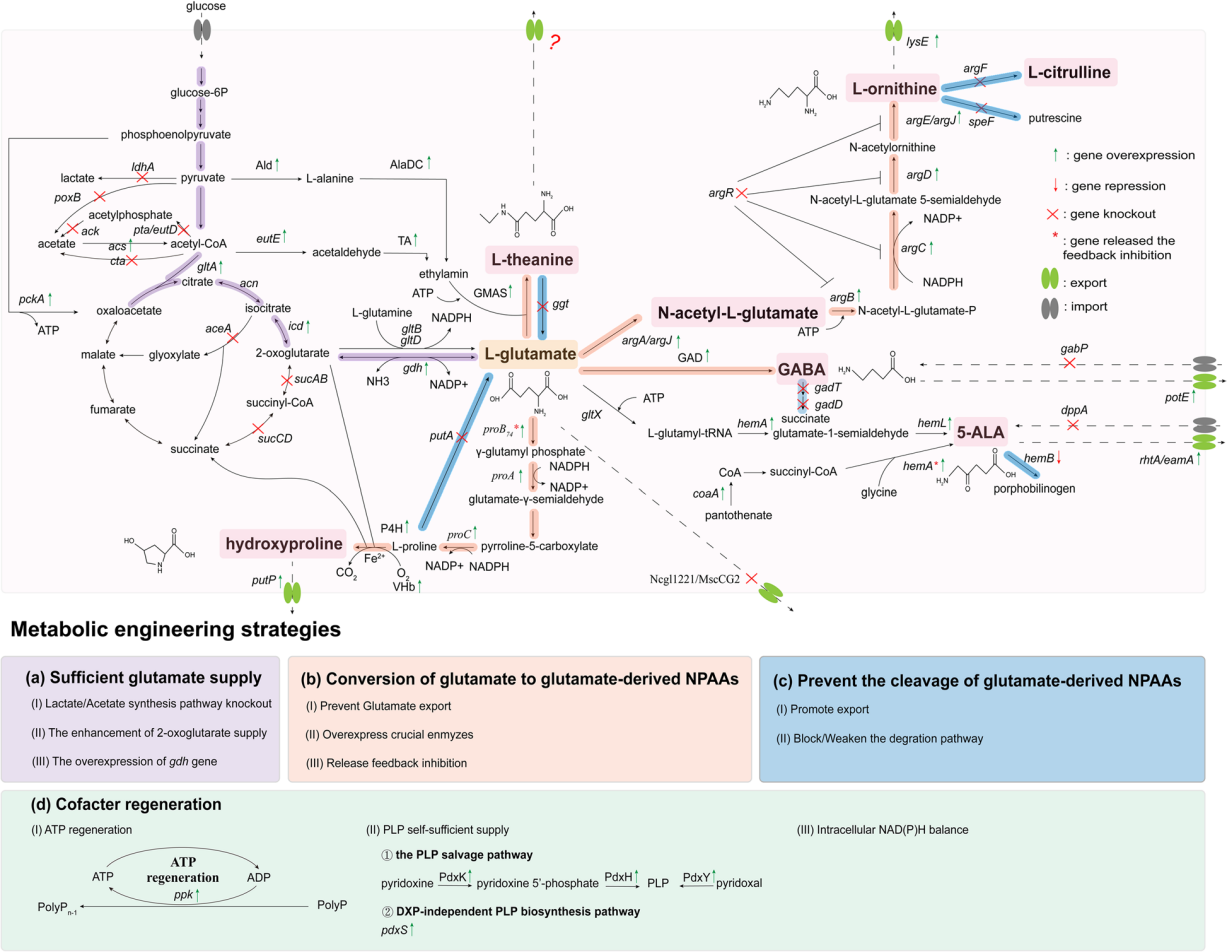
Overview of metabolic pathway, key modification, and engineering strategies of glutamate-derived NPAAs. Ald, alanine dehydrogenase; AlaDC, alanine decarboxylase; *eutE*, encoding aldehyde dehydrogenase; GMAS, γ-glutamylmethylamide synthetase; GAD, glutamate decarboxylase; *gltX*, encoding glutamyl-tRNA synthetase; *hemA*, encoding glutamyl-tRNA reductase; hemA^*^, encoding 5-aminolevulinic acid synthetase; *hemL*, encoding glutamine-1-semialdehyde-2,1-aminomutase; PdxY, pyridoxal kinase; PdxK, pyridoxine kinase; PdxH, pyridoxine 5’-phosphate oxidase; *pdxS*, encoding PLP synthase; *pdxT*, encoding glutamine amidotranferase; P4H, proline-4-hydroxylase; *ppk*, encoding polyphosphate kinase, *proB*_*74*_, encoding feedback resistant γ-glutamate kinase; TA, transaminase; VHb, *Vitreoscilla* haemoglobin

The key metabolic engineering strategies of glutamate-derived NPAAs are systematically summarized in this review. First, glutamate, as an essential amino acid precursor for the biosynthesis of these compounds, is crucial to increase yield through adequate supply. In addition, the enhancement of the metabolic flux from glutamate to target product is equally critical. Simultaneously, strategies to prevent the target product degradation and ensure its timely export from cells further increase yields. Notably, optimizing the intracellular cofactor supply and energy generation are vital to maintain high level of the metabolic pathways to target products and balance cell metabolism with production. Figure 2 illustrates the metabolic pathway and key modification for biosynthesis of five representative glutamate-derived NPAAs.

### Enhancing the supplementation of glutamate

Figure 2 highlights the increasing of glutamate supplementation by reconstructing the carbon flux of Embden-Meyerhof-Parnas pathway (EMP pathway) and tricarboxylic acid cycle (TCA cycle), which is one of the most representative strategies for enhancing the biosynthesis of glutamate-derived NPAAs. This involves: (1) promoting the conversion of pyruvate to acetyl-CoA by preventing lactate and acetate accumulation; (2) enhancing the carbon metabolic flow from oxaloacetate to 2-oxoglutarate; and (3) promoting the conversion of 2-oxoglutarate to glutamate by overexpressing *gdh* gene. The details of metabolic engineering strategies and yield of glutamate-derived NPAAs is shown in Table 1.

**Table 1:** Major advances of glutamate-derived NPAAs production using engineered strains

Products	Strains	Strategies	Titer, g/L	Reactor, L	References
L-Theanine	*E. coli* W3110S3GK	Screen and introduce heterologous alanine dehydrogenase and alanine decarboxylase; Enhance the metabolic flow form acetyl-CoA to ethylamine; Decrease L-theanine degradation	18.7	3	Hagihara et al. [60]
	*Pseudomonas putida* KT2440	Introduce heterologous GMAS and xylose using enzymes; Optimize the selection of carbon sources	21	3.7	Benninghaus et al. [61]
	*C. glutamicum* G01	Screen and engineer GMAS; Strengthen the supply of glutamate; Decrease L-theanine degradation; Enhance intracellular ATP regeneration	44.12	5	Yang et al. [62]
L-Ornithine	*B. amyloliquefaciens* NB	Enhance the supply of glutamate; Weaken the branch pathway; Prevent L-ornithine degradation; Promote L-ornithine export	31.3	7.5	Zhu et al. [63]
	*C. crenatum* SYPA5-5	Block the competing branch of the pathway; Release ornithine acetyltransferase feedback inhibition for L-ornithine; Enhance the carbon flux from glutamate to L-ornithine	40.4	5	Shu et al. [64]
	*C. glutamicum* SO26	Strengthen the supply of glutamate; Optimize the carbon flux of the TCA cycle	43.6	15	Zhang et al. [65]
	*C. glutamicum* MTL13	Strengthen the fructose metabolic pathway; Optimize the selection of carbon sources	54.56	5	Sheng et al. [66]
	*C. glutamicum* SO30	Strengthen the fructose metabolic pathway; Optimize the selection of carbon sources	78.0	5	Nie et al. [67]
	*C. glutamicum* SNK118	Release the feedback inhibition of L-ornithine biosynthesis from glutamate; Block the degradation of L-ornithine; Increase the conversion of glucose to glutamate	88.26	10	Dong et al. [68]
	*C. glutamicum* MTL25	Gene expression regulation based on transcriptomic data	93.6	5	Nie et al. [69]
GABA	*C. glutamicum* ATCC 13032	Optimize GAD; Reconstruct the TCA cycle; Block GABA catabolism; Promote GABA export	23.07	5	Zhang et al. [70]
	*C. glutamicum* ATCC 13032	Strengthen the supply of glutamate; Optimize the heterologous expression of GAD; Weaken branch pathway, block acetate and lactate production; Block GABA catabolism; Promote GABA export	33.17	2.4	Yao et al. [71]
	*C. glutamicum* ATCC 13032	Optimize the heterologous expression of GAD	116	5	Rao et al. [72]
	*E. coli* BW25113	Optimize GAD; Introduce the central regulator of the acid resistance system; Introduce the DXP-independent PLP biosynthesis pathway	307.5	5	Yang et al. [73]
	*E. coli* BW25113	Optimize the heterologous expression of GAD	614.15	200	Ke et al. [74]
	*Kluyveromyces marxianus* C21	—	4.31	0.25	Zhang et al. [75]
	*L. plantarum K16*	—	0.21	0.25	Diez-Gutierrez et al. [76]
	*L. plantarum* KCTC 3103	—	0.67	5	Kim et al. [77]
GABA	*Bifidobacterium adolescentis* JCM 1275	Optimize the expression of GAD; Strengthen the expression of glutamate GABA antiporter	42	0.1	Altaib et al. [78]
	*Lactobacillus hilgardii* GZ2	—	239	5	Zou et al. [79]
	*L. brevis* CD0817	—	321.9	10	Jia et al. [80]
5-ALA	*E. coli* DH5α	Strengthen C_5_ pathway; Accelerate the export of 5-ALA	4.13	5	Kang et al. [81]
	*E. coli* DH5α	Strengthen C_5_ pathway; Decrease 5-ALA degradation; Accelerate the export of 5-ALA	1.78	0.3	Li et al. [82]
	*E. coli* MG1655	Gene integration; Strengthen C_5_ pathway; Plasmid-free production	4.55	0.25	Cui et al. [83]
	*E. coli* BW25113	Strengthen the antioxidant defense system of strain; Introduce C_4_ pathway	11.5	5	Zhu et al. [84]
	*E. coli* DH5α	Decrease 5-ALA degradation; Strengthen C_5_ pathway	1.997	5	Su et al. [85]
	*E. coli* MG1655	Decrease 5-ALA degradation; Weaken branch pathway; Strengthen the supply of succinyl-CoA; Release heme feedback inhibition for 5-ALAS; Accelerate the export of 5-ALA; Strengthen the antioxidant defense system of strain	30.7	5	Pu et al. [86]
5-ALA	*S. oneidensis* MR-1	Introduce C_4_ pathway; Strengthen C_5_ pathway; Strengthen the antioxidant defense system of strain; Decrease 5-ALA degradation; Enhance the carbon flux towards 2-oxoglutarate accumulation in the TCA cycle	1.126	-	Wu et al. [87]
	*E. coli* DH5α	Establish a high-throughput screening method based on the relationship between ROS and cAMP; Directed evolution of 5-ALAS; Enhance the carbon flux towards succinyl-CoA; Decrease 5-ALA degradation; Strengthen the antioxidant defense system of strain	58.54	5	Wang et al. [88]
	*E. coli* Nissle 1917	Introduce C_4_ pathway; Introduce a simultaneous CO_2_ fixation system	1.66	0.1	Effendi et al. [89]
	*E. coli* Rosetta (DE3)	Screen efficient 5-ALAS; Decrease 5-ALA degradation; Enhance the carbon flux from succinyl-CoA to 5-ALA; Block glycine degradation; Accelerate the export of 5-ALA; Dynamically regulate the redox state of strain	63.39	5	Zhou et al. [90]
Hydroxyproline	*E. coli* W3110	Optimize the heterologous expression of P4H; Enhance the supply of 2-oxoglutarate and L-proline; Introduce *Vitreoscilla* haemoglobin	49.8	5	Chen et al. [91]
	*E. coli* W3110	Weaken the degradation of 2-oxoglutarate; Block the degradation of L-proline; Optimize the expression of P4H; Balance the gene expression levels of 2-oxoglutarate and L-proline biosynthesis pathway	8.8	0.25	Zhang et al. [92]
	*E. coli* W3110	Release proline feedback inhibition for γ-glutamyl kinase; Block branch pathway; Increase metabolic flow of 2-oxoglutarate in the TCA cycle; Introduce the non-oxidative glycolysis pathway to produce acetyl-CoA; Enhance the supply of NADPH	89.4	5	Gong et al. [93]

To engineer GABA production by *Corynebacterium glutamicum*, Yao et al. [71] inactivated partial acetate and lactate synthetic pathway by knocking out five genes *eutD*, *aldB*, *poxB*, *lldD* and *ldh*. The attenuation of acetate and lactate biosynthesis pushes the carbon metabolic flux into TCA cycle and glutamate biosynthesis, thereby GABA titer increased 20.4% to 22.47 g/L in *C. glutamicum*. Furthermore, Zhang et al. [70] reconstructed the carbon flux of TCA cycle by overexpressing the *acn* and *icd* genes while disrupting the *sucCD* gene. This approach facilitated great flux conversion from citrate to 2-oxoglutarate thereby strengthening the supply of glutamate for GABA production. Similarly, increasing the metabolic flow of 2-oxoglutarate is beneficial to 5-ALA and hydroxyproline biosynthesis [93, 94]. Wu et al. [87] improved the carbon flux towards 2-oxoglutarate for 5-ALA production by suppressing the *sucA* gene expression. Zhang et al. [92] increased the metabolic flow of 2-oxoglutarate within the TCA cycle by promoting the carbon flux from acetyl-CoA to 2-oxoglutarate and preventing the conversion of 2-oxoglutarate to succinate. Meanwhile, the weaken of acetate and lactate biosynthesis promotes the carbon flux towards TCA cycle, also benefits 5-ALA and hydroxyproline biosynthesis [87, 93]. For L-ornithine production, Zhang et al. [65] enhanced glucose utilization by overexpressing the *iolT* gene, and then push the carbon flux towards TCA cycle and glutamate accumulation by weakening the acetate synthetic pathway. Furthermore, overexpressing *gdh* gene, which catalyzes the conversion of 2-oxoglutarate to glutamate, was shown to positively enhance the biosynthesis of L-theanine, L-ornithine, and GABA [10, 62, 71, 95].

### Modulating the metabolic flow from glutamate to glutamate-derived NPAAs

As a crucial precursor, the supply of glutamate is important to the biosynthesis of glutamate-derived NPAAs. Preventing glutamate export by deleting *Ncgl1221*/*MscCG2* gene is positive to increase the biosynthesis of L-theanine and L-ornithine [62, 65], which would be efficient for the biosynthesis of other glutamate-derived NPAAs.

In nature, 5-ALA is synthesized through two primary pathways, which are designated as the C_4_ and C_5_ pathways. The C_5_ pathway, present in higher plants, algae, and most bacteria, involves a three-step reaction process from glutamate to 5-ALA. Overexpression of the *hemA* and *hemL* genes represents an efficient strategy for 5-ALA biosynthesis through C_5_ pathway [81, 82, 85, 87]. Notably, Cui et al. [83] enhanced the metabolic flow from glutamate to 5-ALA by integrating *hemA* and *hemL* into *E. coli* MG1655, firstly achieving plasmid-free 5-ALA production with 4.55 g/L.

The conversion of glutamate to L-theanine involves only one-step catalyzation by GMAS [10]. Consequently, its activity and catalytic efficiency are crucial to L-theanine biosynthesis. Yang et al. [62] engineered a GMAS from *Paracoccus aminovorans* and directly improved L-theanine yield by 36.61%. Similarly, GABA synthesis from glutamate is one-step catalyzed by GAD. To enhance the catalytic efficiency of GAD, site-directed mutagenesis was performed on GAD derived from *Lactiplantibacillus plantarum* GB01-21. This modification increased the relative enzyme activity of GAD at pH 6.5 from 38% to 84%. This mutated GAD was then heterologous expressed in *C. glutamicum* ATCC 13032, resulting in a GABA titer of 116 g/L [72]. Altaib et al. [78] optimized the expression of GAD and glutamate GABA antiporter (encoded by *gadC* gene), successfully improving the conversion ratio of glutamate to GABA from 84% to 97%.

For L-ornithine biosynthesis, Zhu et al. [63] showed that activating the arginine biosynthetic pathway promotes L-Ornithine biosynthesis by overexpressing *argA* and *argE* genes in *Bacillus amyloliquefaciens*. Furthermore, releasing the feedback inhibition of L-ornithine/hydroxyproline biosynthesis pathway is an efficient strategy to increase the conversion ratio of glutamate to L-ornithine/hydroxyproline. Zhang et al. [65] released the feedback regulation of operon *argCJBD* by deleting the *argR* gene. The mutant gene *proB*_*74*_ alleviates feedback inhibition caused by L-proline accumulation, thereby enhancing hydroxyproline production. Furthermore, the heterologous expression of P4H from *Dactylosporangium* sp., combined with overexpression of the *proA* and *proC* genes, is beneficial to hydroxyproline biosynthesis [91–93, 96].

### Preventing the cleavage of glutamate-derived NPAAs

Accelerating product export is a critical strategy for maintaining low cellular pools of NPAAs, which can prevent its degradation and promote its accumulation. Figure 2 presents the related genes that encode glutamate-derived NPAA transporters. However, the transporter of L-theanine remains notably unclear. To minimize GABA degradation and promote its cellular export, Yao et al. [71] successfully increased GABA yield from 6.2 g/L to 12.71 g/L through knocking out the *gabTDP* gene in *C. glutamicum* ATCC 13032. 5-ALA is a key intermediate of heme biosynthesis, the weakness of the metabolic flow from 5-ALA to heme is positive to increase 5-ALA accumulation. Down-regulating the transcription of *hemB* and *hemF* genes in the heme biosynthesis pathway is beneficial to 5-ALA biosynthesis [82, 85, 97, 98]. For L-ornithine biosynthesis, the catabolism pathway of L-ornithine could be blocked by deleting the *argF* and *speF* genes, thereby preventing its degradation [63, 65, 68, 95]. Furthermore, Yang et al. [62] enhanced L-theanine stability by deleting the *ggt* gene, preventing the conversion of L-theanine to glutamate.

### Improving the intracellular cofactor supply and energy generation

ATP is necessary for both cell growth and NPAA synthesis. As cofactor, ATP directly participates in the transmission of energy to influence the yield of 5-ALA. In the biosynthesis of 5-ALA by C_5_ pathway, the reaction catalyzed by GluRS is ATP dependent [99]. Zhang et al. [100] overexpressed the *pckA* gene to promote the direct conversion of phosphoenolpyruvate to oxaloacetate and ATP generation, finally increased the production of 5-ALA by 12.7%. In addition, the conversion of glutamate to L-theanine and to L-ornithine, both require the addition of ATP. Yang et al. [62] overexpressed the *ppk* gene to promote ATP regeneration during L-theanine production, resulting in the enhancement of intracellular ATP supply.

In the biosynthesis pathway of GABA, the reaction catalyzed by GAD requires pyridoxal 5′-phosphate (PLP) as a cofactor and releases a molecule of CO_2_. Altaib et al. [78] showed that timely supplementation of optimal cofactor PLP in the fermentation medium could restore GAD activity and enhance GABA production. However, the cost of exogenous PLP supplementation remains a challenge. To address this, Yang et al. [73] introduced a PLP biosynthesis pathway independent of 1-deoxy-D-xylulose-5-phosphate (DXP), establishing a self-sufficient PLP system with improved GABA titers. These metabolic modifications significantly enhanced microbial GABA production capabilities through final optimization of fermentation. Similarly, Zhang et al. [97] revealed that strengthen the native biosynthesis pathway of PLP by overexpressing the *pdxH* gene, successfully increasing 5-ALA production by 6.72%.

NADPH provides reducing power for the growth and metabolism of various organisms. In *C. glutamicum*, the biosynthesis of L-ornithine requires 2 mol of NADPH per mol of L-ornithine from glucose substrate. Dong et al. [68] enhanced the supply of NADPH by overexpressing the *rocG* gene from *Bacillus subtilis* or the *gapC* (encoding NADP-dependent glyceraldehyde 3-phosphate dehydrogenase) gene from *Clostridium saccharobutylicum* DSM 13864, resulting in increased production of L-ornithine by 22.1% or 28.8%. Furthermore, maintaining the balance between NAD(P)H and NAD(P)^+^ is necessary for cell growth and production. For the hydroxyproline biosynthesis, Gong et al. [93] maintained the intracellular NAD(P)H balance by enhancing the expression of *PntAB* (the membrane-bound pyridyl nucleotide transhydrogenase in *E. coli*), which is oxidized by NADH to NAD^+^ to drive the reduction of NADP^+^ to NADPH. In the 5-ALA biosynthesis, Zhou et al. [90] achieved an artificial homeostasis of intracellular NADH/NAD^+^ ratio through the heterologous expression of *noxE* (encoding water-forming NADH oxidase), which catalyze the oxidation of NADH to NAD^+^.

## Biosynthesis of aspartate-derived NPAAs

**Fig. 3:**
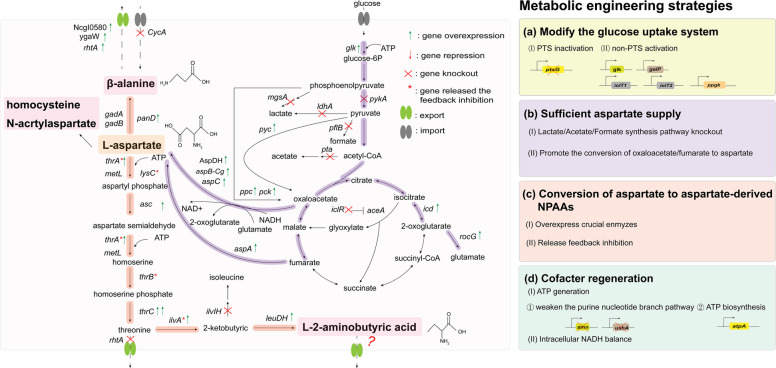
Overview of metabolic pathway, key modification, and engineering strategies of aspartate-derived NPAAs. *amn* (encoding AMP nucleosidase); *aspA* (encoding aspartate ammonia-lyase); *aspB-Cg* (encoding aspartate aminotransferase from *C. glutamicum*); *aspC* (encoding aspartate transaminase); AspDH, aspartate dehydrogenase; *atpA* (encoding ATP synthase); *galP* (encoding D-galactose permease symporter); *glk* (encoding glucose kinase); *iclR* (encoding isocitrate lyase inhibitor); *ilvA* (encoding threonine dehydratase); *ilvIH* (encoding acetohydroxy acid synthase III); *iolT* (encoding myo-inositol permease); *ldhA* (encoding D-lactate dehydrogenase); *leuDH* (encoding dehydrogenase); *lysC* (encoding aspartokinase III); *mgsA* (encoding methyl vinyl ketone synthase); *panD* (encoding L-aspartate-α-decatboxylase); *pflB* (encoding pyruvate-formate lyase); *ppc/pck* (encoding phosphoenolpyruvate carboxylase); *pta* (encoding phosphate acetyltransferase); *ptsG* (encoding IIBC); *pyc* (encoding pyruvate carboxylase); *pykA* (encoding pyruvate kinase II); *rocG* (encoding NADH-dependent glutamate dehydrogenase); *thrA* (encoding aspartokinase I); *ushA* (encoding bifunctional UDP-glycohydrolase)

The key metabolic engineering strategies of aspartate-derived NPAAs are systematically summarized in this review. Firstly, modifying the phosphotransferase system (PTS) to decrease the consumption of phosphoenolpyruvate. Secondly, aspartate, as an essential amino acid precursor for the biosynthesis of these compounds, is crucial to increase NPAA yield. Thirdly, enhancing the metabolic flux from aspartate to target product and weakening the branch pathway are both vital strategies. Finally, improving the intracellular cofactor supply and energy generation are significant in preventing obstruction of the metabolic pathways to target products and balancing cell metabolism and production. Figure 3 shows the metabolic pathway and key modifications of five symbolic aspartate-derived NPAAs. The details of metabolic engineering strategies of aspartate-derived NPAAs are shown in Table 2.

**Table 2:** Major advances of aspartate-derived NPAAs production using engineered strains

Products	Strains	Strategies	Titer, g/L	Reactor, L	Reference
β-Alanine	*P. pastoris* GS115	Optimize the heterologous expression of ADC; Strengthen the supply of aspartate	5.6	1	Miao et al. [101]
	*E. coli* W3110	Optimize the heterologous expression of ADC; Weaken branch pathway of aspartate; Strengthen the supply of aspartate; Enhance the carbon flux towards TCA cycle	43.12	5	Zou et al. [102]
	*E. coli* W3110	Screen ADC and aspartate aminotransferase; Strengthen the supply of oxaloacetate and aspartate; Dynamic regulate the competitive pathways, reduce the loss of aspartate	57.13	5	Zhou et al. [103]
	*E. coli* BW25113	Optimize the heterologous expression of ADC; Enhance the carbon flux of the glyoxylate-TCA cycle; Strengthen the supply of oxaloacetate; relieve the oxidative stress	72.05	1	Miao et al. [104]
	*E. coli* W3110	Optimize the heterologous expression of ADC; Modify the endogenous glucose assimilation system to maintain the flux of phosphoenolpyruvate; Strengthen the supply of oxaloacetate and aspartate; Accelerate the export of β-alanine	74.03	10	Zhang et al. [105]
	*E. coli* W3110	Optimize the heterologous expression of ADC; Reduce the loss of pyruvate and phosphoenolpyruvate; Strengthen the supply of aspartate; Introduce pyruvate carboxylase; Knock out the active transporter of β-alanine, prevent the entry of β-alanine into cells	85.18	5	Li et al. [106]
	*C. glutamicum* ATCC13032	Screen ADC; Introduce the phosphotransferase system (PTS)-independent glucose uptake system; Strengthen the supply of oxaloacetate and aspartate; Accelerate the export of β-alanine	166.6	5	Ghiffary et al. [107]
L-2-Aminobutyric acid	*E. coli* W3110	Release threonine feedback inhibition for aspartokinase I and III; Strengthen the supply of 2-oxoglutarate; Block branch pathway; Release isoleucine feedback inhibition for threonine dehydratase; Promote the conversion of threonine to L-2-aminobutyric acid	9.33	5	Xu et al. [108]
	*E. coli* W3110	Repair the growth defects of chassis strain; Modify the glucose uptake system; Modify the metabolic flow from phosphoenolpyruvate to oxaloacetate; Strengthen the supply of aspartate; Strengthen the supply of ATP and NAD; Improve osmotic resistance	42.14	5	Xu et al. [109]

### Modifying the glucose uptake system

The PTS boosts glucose assimilation and energy regeneration, accompanied by the consumption of phosphoenolpyruvate and overflow of acetate. However, phosphoenolpyruvate is one of the essential precursors for aspartate-derived NPAA synthesis. Therefore, the overexpression of PTS-independent glucose uptake system was reported to be more beneficial for aspartate-derived NPAA synthesis.

Zhang et al. [105] weakened the PTS-dependent system by deleting the *ptsG* gene and overexpressed the *galP* and *glk* genes to recruit an ATP-dependent glucose uptake system. As a result, the β-alanine production increased 120.78%. Similarly, Ghiffary et al. [107] increased the β-alanine production to 4.5 g/L by optimizing PTS-independent glucose uptake system. For L-2-aminobutyric acid biosynthesis, Xu et al. [109] showed the deletion of the *ptsG* gene and overexpression of the *galP* gene successfully balance the circulating flux into EMP pathway, exhibited positive potential in L-2-aminobutyric acid biosynthesis.

### Enhancing the supplementation of aspartate

As an important precursor of aspartate biosynthesis, the supply of oxaloacetate is crucial to aspartate accumulation. On the one hand, promoting the direct conversion of phosphoenolpyruvate/pyruvate to oxaloacetate is effective. The overexpression of *ppc*, *pck*, and *pyc* genes was demonstrated to be effective for β-alanine and L-2-aminobutyric acid biosynthesis [101–103, 106–109]. On the other hand, preventing the overflow of lactate, acetate and formate is beneficial for aspartate-derived NPAA biosynthesis. Zou et al. [102] blocked the metabolic bypass of pyruvate and acetyl-CoA, successfully increased β-alanine production by enhancing the circulating flux into TCA cycle. In addition, promoting the conversion of oxaloacetate/fumarate to aspartate is significant. As shown in Fig. 3, the overexpression of *aspA*, *aspC*, *aspB* genes and AspDH was reported to be positive for increasing aspartate-derived NPAA biosynthesis.

### Modulating the metabolic flow from aspartate to aspartate-derived NPAAs

The conversion of aspartate to β-alanine involves only one-step catalyzation by L-aspartate-α-decarboxylase (ADC). Consequently, enhancing the catalytic efficiency or expression of ADC is the most common method to boost β-alanine biosynthesis. Recently, the heterologous expression of *panD* from *B. subtilis*, which directly catalyzes the conversion of aspartate to β-alanine, has been widely adopted as an effective strategy [101, 103, 107].

For L-2-aminobutyric acid biosynthesis, the conversion of aspartate to L-2-aminobutyric acid involves seven steps. Therefore, minimizing bypass losses and releasing feedback inhibition are feasible strategies to boost L-2-aminobutyric acid production. Xu et al. [108] blocked the metabolic pathways of lysine, methionine, glycine, and isoleucine, and released the feedback inhibition of threonine and isoleucine, finally achieving a L-2-aminobutyric acid titer of 9.33 g/L in a 5-L bioreactor.

### Improving the intracellular cofactor supply and energy generation

ATP plays an important role in the biosynthesis of aspartate-derived NPAAs. Xu et al. [109] increased L-2-aminobutyric acid production through weakening the purine nucleotide branch metabolism by deleting the *amn* and *ushA* genes, and increasing ATP supply by overexpressing the *atpA* gene. In addition, the NADH supply significantly influences biosynthesis of the aspartate-derived NPAAs, as the transamination of oxaloacetate to aspartate requires glutamate conversion and NADH oxidation. In *E. coli*, the intracellular balance of NADH was maintained by replacing the native *gdhA* (encoding glutamate dehydrogenase) gene with the *rocG* gene from *B. subtilis*, which preferentially utilizes NADH rather than NADPH [107]. Simultaneously, overexpressing the *aspC* gene could effectively increase β-alanine production [105]. Furthermore, Xu et al. [109] demonstrated that the enhancement of NAD biosynthesis through overexpressing *pncB* (encoding nicotinamide phosphoribosyltransferase), *nadD* (nicotinamide mononucleotide adenylyltransferase), and *nadE* (NAD^+^ synthetase), could effectively increase L-2-aminobutyric acid production.

## Biosynthesis of other-derived NPAAs

**Fig. 4:**
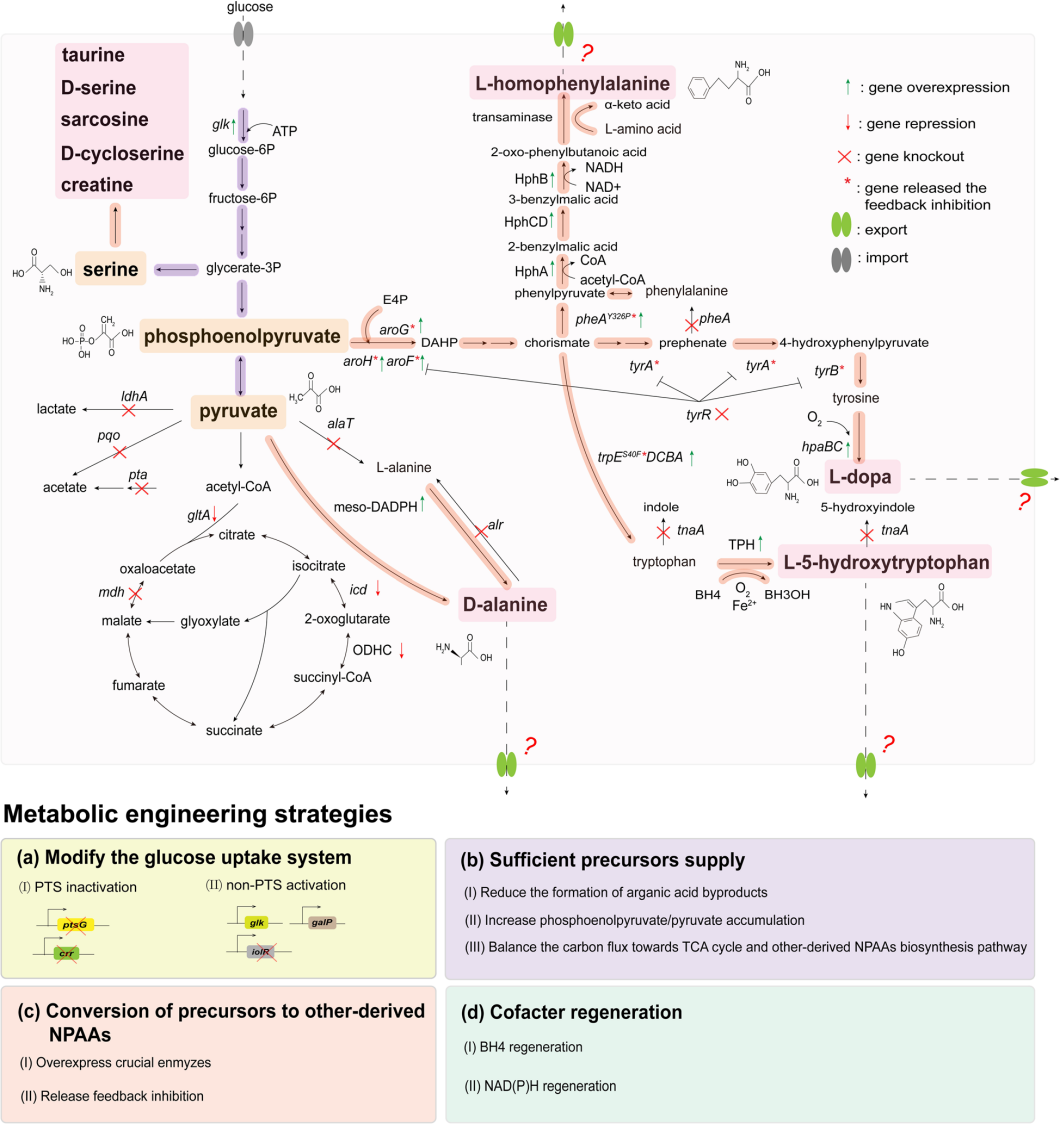
Overview of metabolic pathway, key modification, and engineering strategies of other-derived NPAAs. *alaT*, encoding alanine dehydrogenase; *alr*, encoding alanine racemase; BH4, tetrahydrobiopterin; *aroG/H/F*, encoding DAHP synthase; BH3OH, pterun-4α-carbinolamine; DAHP, 3-deoxy-D-alabino-heptulosonate-7-phosphate synthase; HphA, 2-isopropylmalate synthase; *hpaBC*, encoding 4-hydroxyphenylacetate 3-monooxygenase; HphCD, 3-isopropylmalate dehydratase; HphB, 3-isopropylmalate dehydrogenase; meso-DAPDH, meso-diaminopimelate dehydrogenase; ODHC, 2-oxoglutarate dehydrogenase complex; 6PGL, 6-phosphate D-glucono-1,5-lactone; E4P, erythrose 4-phosphate; *pheA*, encoding prephenate dehydratase; *tnaA*, encoding tryptophanase; TPH, L-tryptophan hydroxylase

Based on the biosynthesis pathway, other-derived NPAAs could be classified as serine-derived, aromatic amino acid-derived, and pyruvate-derived NPAAs. Biosynthesis of other-derived NPAAs from different crucial precursors (serine, phosphoenolpyruvate, and pyruvate) employs diverse metabolic engineering strategies, including the supply of crucial precursors and cofactors, and the block of competitive pathways. The progress of research on three aromatic amino acid-derived NPAAs (L-homophenylalanine, L-dopa, L-5-hydroxytryptophen) and one pyruvate-derived NPAAs (D-serine) have been summarized to show the metabolic pathway and modification of other-derived NPAAs. Figure 4 shows the metabolic pathway and modification of four symbolic other-derived NPAAs. The details of metabolic engineering strategies of other-derived NPAAs are shown in Table 3.

**Table 3:** Major advances of other-derived NPAAs production using engineered strains

Products	Strains	Strategies	Titer, g/L	Reactor, L	Reference
L-Dopa	*Bacillus licheniformis* CICM B6902	Enhance the catalytic efficiency of TH; Strengthen the supply of BH4; Modulate the transcription of shikimate pathway	1.29	15	Xu et al. [110]
	*E. coli* BL21 (DE3)	Release *tyrR* feedback inhibition; Modify the glucose uptake system; Block the phenylalanine biosynthesis pathway; Enhance the supply of phosphoenolpyruvate and E4P; Enhance the catalytic efficiency of *hpaB*	25.53	5	Fordjour et al. [111]
L-5-Hydroxytryptophan	*E. coli* BL21 (DE3)	Strengthen the supply of tryptophan; Promote the conversion of tryptophan to L-5-hydroxytryptophan	1.16	Shake-flask	Xu et al. [112]
	*E. coli* BL21 (DE3)	Construct the BH4 regeneration pathway; Strengthen the supply of tryptophan; Promote the stable expression of TPH	5.1	10	Wang et al. [113]
	*E. coli* W3110	Enhance the catalytic efficiency of TPH; Strengthen the supply of BH4 and tryptophan; Promote NAD(P)H regeneration;	8.58	5	Zhang et al. [114]
D-Alanine	*C. glutamicum* ATCC13032	Block the L-alanine biosynthesis pathway; Strengthen glucose assimilation and phosphoenolpyruvate supply; Optimize the expression of meso-DADPH	85	5	Tian et al. [115]
L-Homophenylalanine	*E. coli* BW25113	Introduce and optimize a BGC for L-homophenylalanine biosynthesis; Block the tyrosine biosynthesis pathway; Release feedback inhibition of *aroG* and *pheA*	1.14	Shake-flask	Liu et al. [116]

### Modifying the glucose uptake system

To increase the accumulation of phosphoenolpyruvate, a major precursor for the biosynthesis of L-dopa, L-5-hydroxytryptophan, L-homophenylalanine, and other NPAAs, the PTS has been a major target for knockout. The introduction of phosphoenolpyruvate-independent glucose transportation system by overexpressing *galP* and *glk* and deleting *pstG* and *crr* could promote phosphoenolpyruvate accumulation and L-dopa production [111]. Tian et al. [115] efficiently increased D-alanine production by integrating the *glk* gene at the *iolR* site, a DeoR transcriptional regulator for activating phosphoenolpyruvate-independent glucose transportation system. Additionally, overexpressing the *tktA* gene (encoding transketolase) effectively enhances the supply of E4P, another crucial precursor for NPAA biosynthesis through shikimate pathway [117].

### Strengthening the supply of crucial precursors

Modulating the EMP pathway is also effective method for the biosynthesis of other-derived NPAAs by reducing the formation of organic acid byproducts and increasing phosphoenolpyruvate/pyruvate accumulation. Tian et al. [115] successfully enhanced pyruvate supply and D-alanine production by introducing the Entner-Doudoroff pathway, which produces pyruvate from glucose in only four steps, while simultaneously reducing carbon flux towards L-lactate and acetate by deleting *ldhA*, *pqo*, and *pta*. Fordjour et al. [111] enhanced phosphoenolpyruvate supply through overexpressing *ppsA* (encoding phosphoenolpyruvate synthase) and deleting *pykF* (encoding pyruvate kinase), effectively promoting tyrosine accumulation and L-dopa biosynthesis.

Notably, balancing the carbon fluxed toward TCA cycle and other-derived NPAAs biosynthesis pathway are essential strategies to improve cell growth and product accumulation. Tian et al. [115] reduced carbon flux to the TCA cycle by weakening the expression of the *gltA*, *icd* and ODHC-encoding genes, effectively enhancing D-alanine production.

### Modulating the metabolic flow from crucial precursors to other-derived NPAAs

As shown in Fig. 4, releasing the feedback inhibition and blocking the competitive pathway have been proved to be effective strategies for the biosynthesis of other-derived NPAAs. The inactivation of the *tyrR* gene has been demonstrated to improve the production of aromatic compounds [111]. In L-dopa biosynthesis, Xu et al. [110] overexpressed feedback-resistant *tyrA* gene (encoding chorismate mutase) to enhance tyrosine accumulation, successfully improved L-dopa production. Furthermore, the biosynthesis of tyrosine and L-dopa production could be increased by knocking out the *pheA* gene to block the phenylalanine biosynthesis pathway [110, 117]. For L-5-hydroxytryptophan biosynthesis, releasing the feedback inhibition of *aroG*, *aroH*, *trpE*, and *serA* (encoding 3-phosphoglycerate dehydrogenase), while deleting the *tnaA* to block indole biosynthesis, are effective targets for tryptophan accumulation and L-5-hydroxytryptophan production [112–114, 118, 119]. For L-homophenylalanine biosynthesis, releasing the feedback inhibition of *aroG* and *pheA*, and deleting the *tyrA* gene exhibit higher phenylalanine and L-homophenylalanine production [116]. However, reducing the accumulation of the precursor L-alanine is significant for increasing D-alanine biosynthesis. Tian et al. [115] deleted the *alaT* and *alr* genes to decrease the formation of L-alanine, which simultaneously promoted the direct transformation from pyruvate to D-alanine and achieved a D-alanine titer of 85 g/L.

### Optimizing the intracellular cofactor supply

The conversion of L-dopa from tyrosine catalyzes by tyrosine hydroxylase requires the participation of cofactor BH4. Similarly, the conversion of L-5-hydroxytryptophan from tryptophan catalyzes by TPH also requires the participation of cofactor BH4. However, the exogenous supplementation of BH4 is expensive. Zhang et al. [114] constructed a BH4 regeneration pathway to promote L-5-hydroxytryptophan biosynthesis. Furthermore, the biosynthesis of BH4 requires NADPH as a cofactor, which could be regenerated by introducing *gdh*_*esi*_ gene (encoding glucose dehydrogenase). Xu et al. [110] successfully enhanced the ability of oxygen transport and NADPH regeneration by overexpressing VHb-encoding gene, with an increase of 37.0% compared to the wild type strain for L-dopa production.

## Conclusions and ​perspectives

### Conclusions

NPAAs exhibit significant potential in enhancing growth, stress resistance, immune function, and early embryonic development, with promising application in agriculture, food, medicine, and cosmetics. Research has also demonstrated their positive effects as dietary supplements, improving exercise performance, reducing physical fatigue, and offering inflammatory, antioxidant, and anti-aging benefits. However, despite extensive studies, the underlying mechanisms behind these functions remain poorly elucidated. Although hundreds of NPAAs have been discovered, their functions and application values remain insufficiently characterized and exploited, with only dozens subjected to comprehensive research. Further studies are required to expand the precise application of NPAAs in nutrition, metabolism, and immunity. Additionally, the value of NPAAs as healthcare products requires deeper exploration.

Advances in metabolic engineering and synthetic biology have significantly improved the microbial production of NPAAs. The engineering strategies employed for NPAAs biosynthesis can be systematically categorized into three key approaches: (1) strengthening the supply of crucial precursors, especially phosphoenolpyruvate, pyruvate, and relevant amino acid precursor. For instance, PTS-independent glucose uptake system was widely used to improve phosphoenolpyruvate accumulation and promote NPAA biosynthesis; (2) blocking the competitive branch pathways from precursor amino acids to NPAAs, while preventing the formation of organic acid byproducts, such as acetate, lactate, etc.; and (3) achieving cofactor self-sufficiency and energy regeneration to maintain redox balance and support high-yield biosynthesis.

### Perspectives

**Fig. 5:**
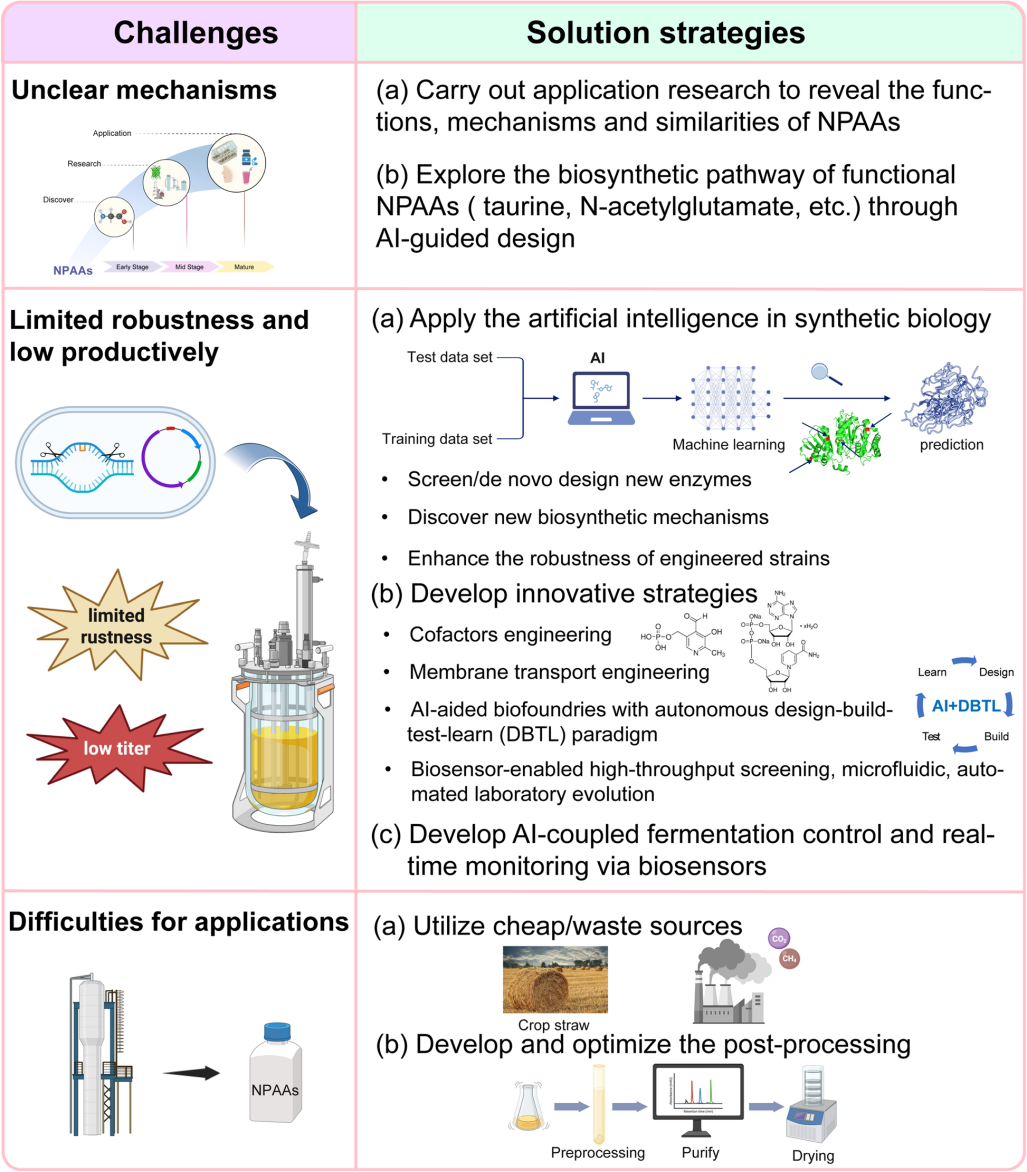
Challenges and potential solution strategies for NPAAs biosynthesis

Currently, several biosynthesized NPAAs, such as GABA, L-theanine and D-alanine, have achieved industrialized application. For example, the biosynthesis of GABA by engineered *E. coli* BW25113 as whole-cell biocatalyst from L-glutamate, has achieved the highest molar yield 99.9%. However, the production of the vast majority of NPAAs remains largely confined to the laboratory scale, facing several critical challenges toward industrial implementation. Production and purification cost are the most important factors to limit their industrial application. For example, to achieve the industrial application of L-5-hydroxytryptophan, more efficient TPH should be identified and the supply of BH_4_ should be self-sufficient. First, biosynthetic and exporting mechanisms of numerous NPAAs remain poorly characterized, such as N-acetylglutamate, taurine, L-2-aminobutyric acid, L-dopa, etc. AI-guided design and pathway prediction tools have great potential to extensive elucidate, design, and optimize the biosynthetic pathways of many functionally important NPAAs. Second, engineered microbial strains must exhibit metabolic robustness to maintain high-yield and high-productivity performance under stressful fermentation conditions. Achieving this will depend on advanced and innovative technologies for enzyme optimization and rewire microbial metabolism, including AI-driven enzyme engineering, biosensor-enabled high-throughput screening, microfluidic, automated laboratory evolution, and AI-aided biofoundries with autonomous design-build-test-learn (DBTL) paradigm. Finally, innovative strategies for the cofactor regeneration, antibiotic-free fermentation, plasmid-free cell factory, and streamlined downstream processing are essential to reduce production cost of NPAA production and enable sustainable biomanufacturing. Future breakthroughs are extremely demanded for the integration of scalable smart technologies, such as AI-coupled fermentation control, real-time monitoring via biosensors, and other novel integrated approaches, to accelerate the translation of NPAA production from bench to industry (Fig. 5).

In the future, the advancement of NPAAs research will be driven by AI-integrated metabolomics, proteomics, and synthetic biology to decode biosynthetic regulatory networks and discover novel functional molecules, such as 5-hydroxytryptamine and cucurbitin. Standard frameworks for application in agriculture must be established to align national strategies for antibiotic reduction and carbon neutrality. The application of these bioproducts in livestock production represents a promising strategy to ​replace antibiotics​ by modulating gut microbiota homeostasis and enhancing host immune resilience, improving feed efficiency and supporting the development of a ​sustainable, low-carbon livestock industry.

## Data Availability

Not applicable.

## Abbreviations

ADC: L-Aspartate-α-decarboxylase
5-ALA: 5-Aminolevulinic acid
AlaDC: Alanine decarboxylase
Ald: Alanine dehydrogenase
AspDH: Aspartate dehydrogenase
BH_3_OH: Pterun-4α-carbinolamine
BH_4_: Tetrahydrobiopterin
DAHP: 3-Deoxy-D-alabino-heptulosonate-7-phosphate synthase
DXP-independent: Oxyxylulose-5-phosphate-independent
EMP pathway: Embden-Meyerhof-Parnas pathway
E4P: Erythrose 4-phosphate
GABA: γ-Aminobutyric acid
GAD: Glutamic acid decarboxylase
GLURS: Glutamyl-tRNA synthetase
GLUTR: Glutamyl-tRNA reductase
GMAS: γ-Glutamylmethylamide synthetase
GSA-AM: Glutamine-1-semialdehyde-2,1-aminomutase
HphA: 2-Isopropylmalate synthase
HphB: 3-Isopropylmalate dehydrogenase
HphCD: 3-Isopropylmalate dehydratase
Hsp: Heat shock protein
meso-DAPDH: Meso-Diaminopimelate dehydrogenase
NPAAs: Nonprotein amino acids
ODHC: 2-Oxoglutarate dehydrogenase complex
PdxH: Pyridoxine 5′-phosphate oxidase
PdxK: Pyridoxine kinase
PdxY: Pyridoxal kinase
6PGL: 6-Phosphate D-glucono-1,5-lactone
P4H: Proline-4-hydroxylase
PLP: Pyridoxal 5′-phosphate
PTS: Phosphotransferase system
TA: Transaminase
TCA cycle: Tricarboxylic acid cycle
TPH: L-Tryptophan hydroxylase
VHb: *V**itreoscilla* haemoglobin
